# A systematic literature review on the clinical efficacy of low dose naltrexone and its effect on putative pathophysiological mechanisms among patients diagnosed with fibromyalgia

**DOI:** 10.1016/j.heliyon.2023.e15638

**Published:** 2023-04-19

**Authors:** Sarah Partridge, Lisa Quadt, Monica Bolton, Jessica Eccles, Charlie Thompson, Alessandro Colasanti, Stephen Bremner, Christopher Iain Jones, Karin Due Bruun, Harm Van Marwijk

**Affiliations:** a Brighton and Sussex Medical School, University of Sussex, UK; b Sussex Partnership NHS Foundation Trust, UK; c Pain Research Centre, Odense University Hospital, Denmark

**Keywords:** Chronic pain, Fibromyalgia, Low dose naltrexone, Systematic review

## Abstract

**Background:**

Low dose naltrexone (LDN) is used off-label by many individuals with fibromyalgia to help manage their pain. There is no current systematic literature review summarising the evidence to support this use of LDN. The objectives of this study were to evaluate if patients with fibromyalgia prescribed LDN have reduced pain scores and greater quality of life compared with those allocated to placebo in randomized controlled trials. Secondly to determine if changes in inflammatory markers and brain structure and function are observed among patients with fibromyalgia taking LDN.

**Methods:**

Systematic literature searches were conducted in MEDLINE**,** Embase Classic + Embase, APA PsychInfo, and The Cochrane Library from inception to May 2022. Reference lists from the selected papers were cross-checked with database search results.

**Results:**

Three studies met our inclusion criteria for the assessment of efficacy, and two studies on potential LDN mechanisms. Results indicated some evidence to suggest LDN reduces pain and increases quality of life. One study reported baseline erythrocyte sedimentation rate (ESR) predicted LDN response (≥30% reduction in fibromyalgia symptoms) and a second study showed plasma concentrations of inflammatory biomarkers were lower after LDN treatment. To our knowledge, there are no brain imaging studies reporting the effect of LDN in patients with fibromyalgia. All studies were based on small sample sizes, were restricted to women and the risk of bias was assessed to be high. There is also some evidence of publication bias.

**Conclusion:**

The strength of evidence from randomized controlled trials to support the use of LDN among patients with fibromyalgia is low. Two small studies suggest ESR and cytokines may be involved in the mechanism by which LDN exerts its effects. Two trials (INNOVA and FINAL) are currently in progress, but further work is needed among men and different ethnic groups.

## Highlights

• There is only low grade evidence to support that LDN reduces pain and improves quality of life in women with fibromyalgia.
• LDN is well tolerated among patients with fibromyalgia in small clinical trials.
• LDN's mechanism of action needs to be further elucidated in human trials.
• Larger robust trials are needed to evidence LDN's efficacy on pain and quality of life associated with fibromyalgia.
• Clinical trials of LDN are needed among men and diverse ethnic groups.

## 1 Introduction

Fibromyalgia is a common condition which can be experienced by anyone and is characterised by widespread pain, stiffness, fatigue, sleep disturbance, cognitive dysfunction and reduced quality of life [[1], [2], [3]]. Estimates of prevalence vary between countries: UK 5.4%, US 3.1%, Europe 2.5% and Asia 1.7% and among specific populations, for example 9% among female textile workers in Turkey and 10% among metalworkers in Brazil [[3], [4], [5], [6]].

Current recommended treatments for fibromyalgia focus on graded exercise, psychological therapies and antidepressants [7]. However, patients with chronic pain report that these are only partially helpful [8,9].

Low dose naltrexone (LDN) is not currently licensed for the management of chronic pain syndromes including fibromyalgia. However, many patients with these conditions access LDN privately and report an improvement in symptoms and quality of life [8]. This is consistent with reports from open-label studies suggesting LDN is associated with reduced pain and improved quality of life among individuals with fibromyalgia [10,11]. Recent systematic reviews reported LDN has a good safety profile [12,13].

There is a need for an updated systematic literature review on the clinical efficacy of LDN and its effects on putative pathophysiological mechanisms among patients diagnosed with fibromyalgia to guide and inform future research and clinical decision making.

In this systematic review we pose three questions: Firstly, we investigate whether LDN is associated with reduced pain scores and greater quality of life in patients with fibromyalgia, compared with those allocated to placebo in clinical trials. Secondly, we ask which (if any) changes in inflammatory biomarkers are observed among fibromyalgia patients taking LDN. Thirdly we explore if there are any reports of changes to brain structure and function among fibromyalgia patients taking LDN.

## 2 Method

This systematic literature review followed the Preferred Reporting Items for Systematic Reviews and Meta-Analyses (PRISMA) guidelines [14] and has been registered with the International Prospective Register of Systematic Reviews (PROSPERO). Registration number: CRD42022312279: Registration date: February 24, 2022.

## 3 Search strategy and selection criteria

The following electronic databases were searched from inception to May 2022: MEDLINE, Embase Classic and Embase, APA PsychInfo, The Cochrane Library (Cochrane Database of Systematic Reviews, Cochrane Central Register of Controlled Trials (CENTRAL), Cochrane Methodology Register). Searches were re-run in August 2022. Reference lists from the selected papers were cross-checked with the database search results. Authors were contacted for additional information where necessary.

The following search strategies were used:

Search strategy 1: To explore the efficacy of LDN among individuals with a diagnosis of fibromyalgia: [fibromyalgia] AND [naltrexone].

Search strategy 2: To explore the effect of LDN on inflammatory biomarkers among individuals with a diagnosis of fibromyalgia: [fibromyalgia] AND [naltrexone] AND [cytokine OR cytokines OR “cytokine signatures” OR “inflammatory cytokines” OR “inflammatory markers” OR CRP OR “c-reactive protein” OR ESR OR “erythrocyte sedimentation rate” OR “plasma viscosity”]

Search strategy 3: To explore the effect of LDN on brain images among individuals with a diagnosis of fibromyalgia: [fibromyalgia] AND [naltrexone] AND [MRI OR “magnetic resonance imaging” OR fMRI OR “functional magnetic resonance imaging” OR PET OR “positron emission tomography”]

Inclusion criteria for LDN efficacy included placebo-controlled intervention trials in humans of any age and with a diagnosis of fibromyalgia (by any recognised diagnostic criteria) who received treatment with oral LDN (4–12 mg per day) or placebo. Observational studies of humans with a diagnosis of fibromyalgia and taking LDN for the assessment of pathophysiology were also included. Studies without a control group, case series and case reports were excluded due to the high potential for bias in these study designs. Animal studies were also excluded.

Eligibility for inclusion in the review was determined by reading the abstracts of each study identified by the search. Studies that did not meet the inclusion criteria were excluded. Full copies of the remaining potential eligible studies were obtained for detailed assessment of eligibility. Information was extracted on study setting, study design, study duration and follow-up, outcome measures, demographic characteristics and number of participants, results, withdrawals and adverse events.

Bias was assessed using the Cochrane Risk of Bias Methodology [15] and quality of evidence was assessed using the GRADE methodology [16].

Two review authors [SP and LQ] independently ran the searches, made decisions on eligibility, extracted the data and conducted the risk of bias assessments. Agreement at each stage was reached through discussion with HM.

## 4 Results 1 – Efficacy of low dose naltrexone among individuals with a diagnosis of fibromyalgia

### 4.1 Results of the searches

**Fig. 1:**
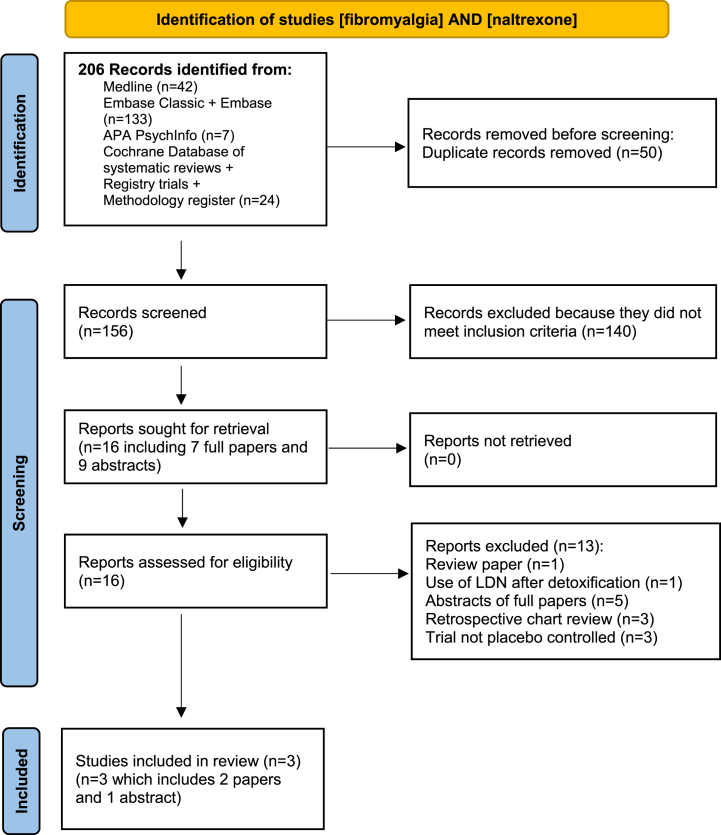
Identification of studies of fibromyalgia and low dose naltrexone.

The database searches yielded 206 records of which 50 were duplicates. The titles and abstracts of the remaining 156 were reviewed. Following this, 140 records were excluded. The full texts of the remaining 16 records were retrieved. No additional novel reports were identified from the reference lists of these records. From the 16 full text articles reviewed, 13 were excluded for the following reasons: 1 was a review article; 1 described the use of LDN after opioid withdrawal; 5 were abstracts that preceded published full papers identified by the search strategy; 3 described retrospective chart reviews and 3 trials were not placebo controlled. The remaining 3 studies (2 full papers and 1 abstract) were included in the review [[17], [18], [19]]. The author of the abstract [19] was contacted for further information but did not respond. See Fig. 1.

### 4.2 Study characteristics

Three placebo-controlled clinical trials involving a total of 117 adult participants were included in the review [[17], [18], [19]]. Two studies were conducted by the same research team in a single centre in the United States [17,18] and one study was conducted in Egypt [19]. Two studies had a cross-over design [17,18] and one had a parallel group design [19]. One study was single-blind, so that only the participants were unaware if they were taking LDN or placebo [17]. Sample-size calculations were not reported [[17], [18], [19]].

In the 2009 Younger study all patients started with a 2 week placebo phase followed by an 8 week drug treatment phase and finished with a 2 week washout phase [17]. In the second cross-over study by Younger, after a 2 week baseline period, patients were randomly allocated to either 4 weeks' placebo followed by 8 weeks’ active treatment or the reverse [18]. There was no washout period between placebo and active treatment. Patients ended the study with a 4 week follow up period where no study drugs were issued. The parallel group study randomized patients to either active treatment or placebo for 24 weeks [19].

The studies in the US were funded by charitable donations [17,18]. The funding for the Egyptian study was not stated [19]. None of the studies reported conflicts of interest.

### 4.3 Participant characteristics

Table 1 shows the key baseline characteristics of the study participants. The ethnicity of participants was not reported. Of the 117 participants recruited, 5 individuals were excluded from the analyses, and thus results were only presented on 112 patients. Reasons given for exclusions included: use of opioid medication, major physical accident, consent withdrawn due to side-effects, consent withdrawn due to use of LDN, computer error resulting in loss of data.

**Table 1:** Patient Characteristics from the 3 trials evaluated to assess LDN Efficacy.

	Younger et al. [17]	Younger et al. [18]	Abou-Raya [19]
Number of participants randomized	12	31	74
Number participants reported in the analysis	10	28	74
Women n (%)	10 (100%)	28 (100%)	Not reported
Age (years) mean (SD, range)	44.0 (±10.3, 22–55)	42.7 (±12.9, 23–65)	Not reported
Duration of fibromyalgia (years), mean (SD, range)	9.6 (±6.5, 1–20)	11.7 (±10.1, 0.7–44)	Not reported
Baseline fibromyalgia Impact Questionnaire, mean (SD, range)	67.2 (±15.0, 39.4–85.8)	57.2 (±11.8, 29–76)	Not reported

All three studies required patients to meet the American College of Rheumatology (ACR) 1990 diagnostic criteria for fibromyalgia [2] and excluded patients using opioid analgesia. Both studies by Younger also excluded patients with evidence of joint pain/inflammation or diagnosis of autoimmune or rheumatologic condition, rheumatoid factor >20 IU/mL, antinuclear antibody >1:80, erythrocyte sedimentation rate >60 mm/h. The Younger 2013 study also excluded patients with *C*-reactive protein level >2 mg/dL, significant psychiatric distress, or Revised Beck Depression Inventory (BDI-II) score >29.

### 4.4 Interventions

All three studies prescribed the active treatment, oral naltrexone 4.5 mg daily. In the study by Younger 2013, 4 patients requested a lower dose due to side-effects and took naltrexone 3 mg. Tablets were taken approximately 1 h before bedtime in both studies by Younger. The timing of the dose was not reported in the study by Abou-Raya.

### 4.5 Measurements

In the Younger 2009 study the primary outcome was change in overall self-reported fibromyalgia symptom severity.

In both the Younger 2013 and Abou-Raya 2013 studies the primary outcome was change in self-reported daily pain between baseline and end of active treatment.

All three studies used the Fibromyalgia Impact Questionnaire (FIQ) score to assess fibromyalgia symptoms and severity.

All participants self-assessed pain and other quality of life measures including fatigue, sleep quality, ability to think and remember, mood, satisfaction with life on a visual-analogue scale with a range of 0–100.

In both studies by Younger, participants recorded self-reported symptoms on a hand-held computer every evening before bedtime and attended a laboratory for clinician-led assessments every 2 weeks. In the study by Abou-Raya, pain was assessed by both patient and clinician.

In both Younger studies, self-reported medication tolerability was measured daily using a 0–100 visual-analogue scale with 0 ‘cannot tolerate at all’ and 100 as ‘tolerate perfectly well”. In the Abou-Raya study safety and tolerability were assessed but the specific method was not described.

### 4.6 Outcomes

#### 4.6.1 Self-reported pain

Self-reported pain was the primary outcome in 2 studies [18,19] and a secondary outcome in 1 study [17].

In the Younger 2013 study, baseline pain was calculated by averaging daily pain across the entire 14-day baseline period and endpoint pain was the average pain score during the final 3 days of treatment. A sensitivity analysis was conducted using data from the final 7 days of treatment. The Abou-Raya study reported pain at baseline and at Week 24.

Mean (± standard deviation [SD]) baseline pain was lower in the Younger 2013 study (50.8, SD not reported), compared with the Abou-Raya study (65.6 ± 13.5 in the LDN group versus 66.0 ± 14.1 in the placebo group). Baseline pain was not reported in the Younger 2009 study.

Reductions in pain during LDN treatment were reported in all 3 studies (Table 2 and Table 3). The Younger 2013 study reported a mean (±SD) percentage reduction in pain of 28.8% ± 9.3% during the last 3 days of LDN treatment compared with 18.0% ± 10.8% during placebo (P = 0.016). Analysis repeated averaging the last 7 days of pain gave similar results, but data was not reported. The Abou-Raya study reported the absolute mean (±SD) pain score in the LDN group as 65.6 ± 13.5 (baseline) and 45.5 ± 14.1 (Week 24) compared with 66.0 ± 13.5 (baseline) and 55.3 ± 13.9 (Week 24) in the placebo group (P < 0.001). The Younger 2009 study did not report absolute values but commented that treatment with LDN reduced both average daily pain and highest reported pain.

**Table 2:** Summary of key results from the efficacy trials.

	Younger et al. [17]	Younger et al. [18]	Abou-Raya [19]
Change in Pain	Authors described LDN was associated with a reduction in average daily pain and highest pain (values not reported)	Percentage reduction in pain (mean ± SD)	Pain score (mean ± SD)
		LDN 28.8% ± 9.3% vs placebo 18.0% ± 10.8%	LDN 65.6 ± 13.5 (baseline) and 45.5 ± 14.1 (Week 24) vs placebo 66.0 ± 13.5 (baseline) and 55.3 ± 13.9 (Week 24)
FIQ score	Mean percentage reduction in overall FIQ score	Change in overall FIQ score not reported. Authors noted LDN associated with improved life satisfaction and mood	FIQ score (mean ± SD) LDN 63.7 ± 13.2 (baseline) and 38.5 ± 16.1 (Week 24) vs placebo 63.0 ± 13.0 (baseline) and 57.9 ± 15.6 (Week 24)
	LDN 31.7% vs placebo 16.7%		
Responders to LDN treatment	6/10 participants reported ≥30% reduction in symptom severity on LDN compared with placebo	9/28 participants on LDN reported both ≥30% reduction in pain and ≥30% reduction in either fatigue or sleep problems during LDN treatment vs 3/28 on placebo	Authors described 16/37 patients on LDN had ‘*clinically important*’ improvements in total FIQ score and quality of life
Adverse events	Adverse events described as mild.	LDN vs placebo: Headaches (16% vs 3%) and vivid dreams (37% vs 13%)	LDN vs placebo: Headaches (15% vs 5%) and vivid dreams (33% vs 14%)
Tolerability of LDN	96.3% of participants described LDN as tolerable vs 89.7% on placebo	Tolerability score (Mean ± SD)	Not reported
		LDN 89.2 ± 15.1 vs placebo 89.4 ± 15.6	

Abbreviations: LDN, low dose naltrexone; SD, standard deviation; FIQ, fibromyalgia impact questionnaire.

**Table 3:** Study results presented with 95% Confidence Intervals*.

	Mean	n	SD	95% CI
**Younger 2013** [18]				
Mean % reduction in pain LDN	28.8	14	9.3	23.4 to 34.2
Mean % reduction in pain placebo	18.0	14	10.8	11.8 to 24.2
Tolerability score LDN	89.2	14	15.1	81.3 to 97.1
Tolerability score placebo	89.4	14	15.6	81.2 to 97.6
**Abou-Raya** [19]				
Pain score LDN baseline	65.5	37	13.5	61.2 to 69.8
Pain score LDN week 24	45.5	37	14.1	41.0 to 50.0
Pain score placebo baseline	66.0	37	13.5	61.7 to 70.3
Pain score placebo week 24	55.3	37	13.9	50.8 to 59.8
FIQ score LDN baseline	63.7	37	13.2	59.4 to 68.0
FIQ score LDN week 24	38.5	37	16.1	33.3 to 43.7
FIQ score placebo baseline	63.0	37	13.0	58.8 to 67.2
FIQ score placebo of week 24	57.9	37	15.6	52.9 to 62.9

*95% Confidence Intervals calculated from data in the original papers.

Abbreviations: n, number participants; SD, standard deviation; 95% CI, 95% confidence interval.

#### 4.6.2 Change in self-reported fibromyalgia symptoms

In the Younger 2009 study the primary outcome was change in overall fibromyalgia symptom severity. LDN was associated with a reduction in overall symptom severity compared with placebo (32.5% versus 2.3%, P < 0.001).

Change in Fibromyalgia Impact Questionnaire (FIQ) total score was the secondary outcome in 2 studies [17,19]. FIQ-rated symptom severity was reduced in both studies Table 2, Table 3. In the Younger 2009 study, the mean percentage reduction in overall FIQ score was 31.7% in the LDN group compared with 16.7% in the placebo group (P < 0.001). The Abou-Raya study reported the absolute mean (±SD) FIQ score in the LDN group as 63.7 ± 13.2 (baseline) and 38.5 ± 16.1 (Week 24) compared with 63.0 ± 13.0 (baseline) and 57.9 ± 15.6 (Week 24) in the placebo group (P < 0.001).

In the Younger 2013 study, the change in overall FIQ score was not reported. The authors reported that LDN compared with placebo was associated with improved life satisfaction (11.1% vs 3.2%, P = 0.045) and improved mood (10.7% vs 2.1%, P = 0.039), Table 2. However, they noted that there was no difference in sleep (10.4% vs 9.2%, P = 0.575) or fatigue (12.6% vs 7.8%, P = 0.461). The Younger 2009 study reported that while LDN had an impact on average daily pain, highest pain, fatigue and stress, no effect was observed for self-reported sleep quality, gastrointestinal problems, headaches, thinking and concentration or sadness.

#### 4.6.3 Responders to LDN treatment

All three studies commented on the number of patients with clinically important improvements in symptoms during LDN treatment (Table 2).

The Younger 2009 study defined a drug responder as a participant with ≥30% reduction in symptom severity during LDN treatment compared with placebo and reported 6/10 participants (60%) met this criterion.

The Younger 2013 study defined a drug responder as a participant with ≥30% reduction in pain and ≥30% reduction in either fatigue or sleep problems. Using these criteria, 9/28 participants (32%) were classified as drug responders during the LDN treatment phase compared with 3/28 (11%) on placebo (P = 0.050). Using the criterion ≥30% reduction in pain alone, 16/28 participants (57%) were drug responders.

The Abou-Raya 2013 study reported 16/37 participants (43%) randomized to LDN had ‘clinically important’ improvements in total FIQ score and quality of life, although the criteria for ‘clinically important’ was not defined.

#### 4.6.4 Tolerability and adverse events

In the studies by Younger 2013 and Abou-Raya 2013 headaches and vivid dreams were more frequently reported in the LDN group compared with the placebo group. In the Younger 2013 study, headaches were reported by 16% in the LDN group and 3% in the placebo group (P = 0.044) compared with 15% and 5% respectively in the Abou-Raya study. In the Younger 2013 study, vivid dreams were reported by 37% in the LDN group and 13% in the placebo group (P = 0.037) compared with 33% and 14% respectively in the Abou-Raya 2013 study (Table 2).

Other side-effects reported in the studies included insomnia, nausea, dry mouth, shortness of breath, anxiety, agitation, increased hair growth, increased sweating, dizziness. No Serious Adverse Events were reported in any of the three studies.

In the study by Younger 2009, all adverse events were described as mild and no change in dosage was required. In the study by Younger 2013, 1 individual dropped out of the study after 3 days due to dry mouth/eyes and tinnitus. 4 participants requested a dose reduction from 4.5 mg to 3 mg due to side-effects. Among those requesting a dose reduction (3 participants were taking LDN and 1 was taking placebo) the severity of side-effects was subsequently reduced.

In the study by Younger 2009, 96.3% of participants described the study drug as tolerable during LDN treatment compared with 89.7% during placebo treatment.

In the study by Younger 2013, LDN was rated equally tolerable as placebo. The mean ± SD tolerability score was 89.2 ± 15.1 during LDN treatment and 89.4 ± 15.6 during placebo (P = 0.809), Table 2, Table 3

## 5 Results 2 – Effect of low dose naltrexone on inflammatory markers among individuals with a diagnosis of fibromyalgia

### 5.1 Results of the searches

**Fig. 2:**
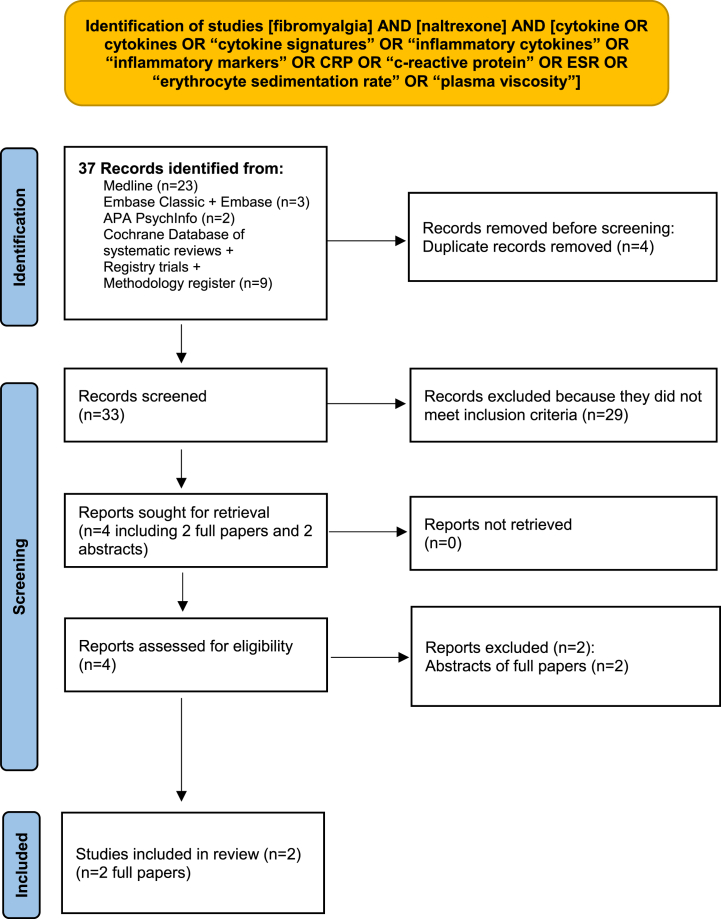
Identification of studies of fibromyalgia, LDN and Inflammatory Markers.

The database searches yielded 37 records of which 4 were duplicates. The titles and abstracts of the remaining 33 articles were reviewed. Following this, 29 records were excluded. The full text of the remaining 4 records were retrieved. No additional novel reports were identified from the reference lists of these records. From the 4 full text articles reviewed, 2 were abstracts which were superseded by published full papers identified by the search strategy and were excluded. Thus 2 studies were included in the review. See Fig. 2.

### 5.2 Younger 2009 study [17]

The study published by Younger in 2009 has previously been described [17]. In brief, this was a single-blind, placebo-controlled cross-over trial. Ten participants with a diagnosis of fibromyalgia according to the ACR 1990 criteria [2] completed the study. Of note, the cross-over sequence was not randomized and all participants received 2 weeks of placebo followed by 8 weeks of 4.5 mg oral naltrexone taken approximately 1 h before bedtime. Table 1 shows the demographic characteristics of the study participants. During the study, participants recorded their symptoms every night and visited the laboratory every 2 weeks.

As previously reported, LDN had an impact on fibromyalgia symptom severity and 6/10 participants met the study criteria for a ‘drug responder’, defined as ≥30% reduction in symptom severity. An individual responder analysis was conducted to determine whether baseline erythrocyte sedimentation rate (ESR), severity of fibromyalgia and duration of illness predicted positive drug response. In this small study, baseline ESR values explained over 82% of the variance in LDN response. The correlation between response and baseline ESR was 0.91, P < 0.001. Those who had the highest baseline ESR levels (noting all patients with a baseline ESR >60 mm/h were not eligible for participation in the study) had the greatest positive response to LDN. Neither duration of illness nor baseline symptom severity predicted response to LDN. Unfortunately, ESR was not measured during or at the end of LDN/placebo treatment, so the authors were unable to comment on whether ESR changed during the study.

### 5.3 Parkitny 2017 study [11]

The study by Parkitny was a 10 week, single-blind trial which included 8 participants with a diagnosis of fibromyalgia as defined by the ACR 2010 diagnostic criteria [11]. Participants had a 2 week baseline period where no tablets were administered followed by 8 weeks of 4.5 mg oral naltrexone taken approximately 1 h before bedtime. Participants were told it was a cross-over design and they could receive placebo at any time during the protocol, but no placebo tablets were administered. Exclusion criteria included regular use of anti-inflammatory medication; known rheumatological/autoimmune condition; baseline temperature >37.8C (100 F); ESR >60 mm/h; c-reactive protein (CRP) > 3.0 mg/dL; Antinuclear antibody (ANA) > 1:80; positive rheumatoid factor. During the study patients recorded symptoms daily before bedtime. Blood samples were taken twice weekly during baseline and active drug treatment phases. Laboratory visits were scheduled within the same 2 h window to minimize bias associated with diurnal cycles of cytokine expression. The primary outcome was the change in 63 plasma biomarkers of inflammation [IL-6, IL-12p70, TNF-α, IL-15, IL-17A, IL-12p40, TGF-β, IL-1Ra, IL-10, IL-27, IL-5, IFN-α, IL-4, IL-1β, IL-2, G-CSF, TGF-α, LIF, ENA78 (CXCL5), IFN-γ, IFN-β, ICAM-1 (CD54), IL-13, MIP-1α (CCL3), Resistin, MCP3 (CCL7), VEGF, NGF, IL-18, SCF, MCP-1 (CCL2), IL-17F, CD40L (CD154), IL-7, MIP-1β (CCL4), VCAM1 (CD106), HGF, Eotaxin (CCL11), TRAIL, GM-CSF, IL-21, PIGF-1, IL-1α, IP-10 (CXCL10), PAI1, EGF, RANTES (CCL5), SDF1a (CXCL12), VEGF-D, Leptin, BDNF, PDGF-BB, MIG (CXCL9), IL-22, IL-31, IL-9, TNF-β, GRO-α (CXCL1), IL-23, MCSF, FGF-β, FasL, IL-8 (CXCL8)] between the 2 week baseline period and the final 2 weeks of LDN administration. Secondary endpoints were change in pain severity and change in overall symptom severity between baseline and drug condition.

Results indicated that plasma concentrations of almost all 63 inflammatory biomarkers were lower after 6 weeks of LDN treatment. In addition, self-reported pain was lowered by 13.9% (P < 0.001) and overall symptoms were reduced by 23.3% (P < 0.001). However, there was no comparator group in this study.

## 6 Results 3 - Effect of low dose naltrexone on brain images among individuals with a diagnosis of fibromyalgia

Our literature searches did not identify any brain imaging studies among patients with fibromyalgia before and after taking LDN. Of interest is the INNOVA clinical trial [20] which was in progress at the time of writing and includes a brain imaging sub-study that aims to further elucidate the mechanism of action of LDN.

## 7 Risk of bias assessed using the Cochrane risk of bias tool

The overall risk of bias using the Cochrane Risk of Bias Tool [15] was assessed to be high in all four studies included in this systematic review.

The study by Abou-Raya [19] was published as an abstract, and despite attempts to contact the author no further details were obtained. There was insufficient information to adequately assess methodological quality and the risk of bias was considered to be high.

The study by Younger 2009 [17] did not report a sample size calculation, had a very small sample size (n = 10), short duration of follow-up, was single-blind and did not randomly allocate participants to a treatment sequence. Furthermore, two participants (17% of the total study sample) were excluded from the analyses. The risk of bias was considered to be high.

The study by Younger 2013 [18] also did not report a sample size calculation, had a small sample size (n = 28) and a short duration of follow-up. There was no washout phase between treatment and placebo in this cross-over design and a large carryover effect may have reduced the observed effect size of LDN. In this study 3 participants (10% of the total study population) were excluded from the analyses. The risk of bias was considered to be high.

The major limitations in the study by Parkitny 2017 [11] were the lack of a control group, the small sample size and the short duration of treatment. Furthermore, a very large number of inflammatory biomarkers (n = 63) were chosen as the primary outcome. Although *P*-values were corrected for multiple comparisons, the number deemed to change significantly (n = 17) is highly dependent upon the set threshold (P = 0.017).

## 8 Publication bias

To our knowledge at the time of writing, two trials of LDN in patients with fibromyalgia have been completed but not published either as a conference abstract or in a peer reviewed journal [21,22]. The first trial, conducted in Denmark, commenced in June 2016 and the estimated completion date was December 2017. This crossover trial was designed to enrol 140 men and women [21]. Personal communication with the authors indicated that the trial has been completed but, to date, has not been submitted for publication. The second trial, conducted in Brazil, started in August 2018 and was completed in July 2020. This parallel group trial enrolled 92 women [22]. Attempts to contact the authors to discuss the study results were unsuccessful. This raises concerns regarding publication bias if trials that did not demonstrate an association between LDN and reduced pain or improved quality of life are not published.

## 9 Quality of evidence assessed using the GRADE approach

The quality of evidence to answer our review questions on LDN efficacy using the GRADE methodology [16] was assessed to be very low and the true effect size may be substantially different from that reported. This is because, to our knowledge at the time of writing, the results of only two small, placebo-controlled trials, each with significant limitations and a total of 43 participants have been published in peer reviewed journals and one study of 74 participants published as a conference abstract. Furthermore 2 studies have been completed but the results have not yet been published [21,22].

The evidence to assess mechanisms of action of LDN among individuals with fibromyalgia is extremely limited, and as a result there is a high level of uncertainty in true effect size.

## 10 Discussion

The findings from this systematic literature review suggest that [1] there is only low grade evidence to support that LDN reduces pain and improves quality of life among women with fibromyalgia [2] LDN was well tolerated in small clinical trials and [3] the effect of LDN on inflammatory markers and brain structure and function needs to be further elucidated in human trials.

The findings on LDN efficacy are drawn from a small number of published, placebo-controlled trials among patients with fibromyalgia. At the time of writing and to the best of our knowledge, there are two published papers and one published abstract meeting these criteria [[17], [18], [19]]. We are also aware of two further completed trials that have not yet been published, raising concerns over publication bias [21,22].

The three published efficacy trials included in this review consistently demonstrated a reduction in self-reported pain among patients with fibromyalgia when treated with 4.5 mg LDN, but were all associated with a high risk of bias [[17], [18], [19]]. The studies also reported the proportion of participants defined as ‘drug responders’ as between 32% and 60%. However, the definitions of ‘drug responder’ varied between the studies making direct comparisons difficult. The studies also reported changes in other symptoms associated with fibromyalgia, including overall reduction in symptom severity and improvement in life satisfaction and mood, but again the trials varied in what outcomes were reported making direct comparison difficult.

All three studies reported adverse events which were described as mild. No serious adverse events were reported. These results are consistent with a systematic literature review and meta-analysis pooling results from 89 trials of naltrexone for alcohol use disorders, other addictions, Crohn's disease, cancer and fibromyalgia which concluded that there was no evidence of increased risk of serious adverse events for naltrexone compared to placebo (Risk Ratio 0.84, 95% Confidence Interval 0.66 to 1.06) [12].

Studies exploring the mechanisms by which LDN exerts its effect suggest that ESR and other inflammatory markers may play an important role [17,11]. The Younger 2009 study reported baseline ESR predicted LDN response albeit in a very small sample (n = 10) [17]. The Parkitny study showed inflammatory biomarkers were reduced after LDN treatment albeit in a study with no control group [11]. A post hoc analysis combining data from both Younger studies reported similar results [23] and was consistent with a study of LDN among patients with Crohn's disease [24].

To our knowledge there are no studies that have reported the impact of LDN on brain structure or function investigated via imaging studies, among patients with fibromyalgia. However, two studies are currently in progress to explore the efficacy and mechanisms by which LDN exerts its effect among patients with fibromyalgia. The INNOVA study [20] is recruiting 120 women in Spain to participate in a randomized, double-blinded, placebo-controlled, parallel design trial of 4.5 mg/day LDN. Patients will be followed up for 1 year. To study mechanisms, a sub-group of patients will undergo MRI scanning for changes in brain metabolites related to neuroinflammation and central sensitisation, and blood tests for serum inflammatory biomarkers. The FINAL study [25] is recruiting 100 women in Denmark to participate in a randomized, double-blind, placebo-controlled, parallel design trial of 6 mg/day LDN. Patients will be followed up for 12 weeks. Exploratory analyses to investigate the mechanisms of action of LDN include measures of pain sensitivity, muscle performance and biomarkers. The results of these trials are awaited with great interest as both studies will further elucidate the efficacy and mechanisms of action of LDN among women with fibromyalgia.

A major limitation of all three efficacy trials was the very small sample sizes, with data on a total of only 112 participants having been analysed, leading to a great deal of uncertainty surrounding the results. Furthermore, the baseline characteristics in the 2 studies by Younger [17,18] showed a wide range in the number of years participants had a diagnosis of fibromyalgia (between less than 1 year and 44 years) and in self-reported fibromyalgia symptom severity (rated between 29 and 86 out of 100). However, an analysis, albeit in a very small sample, reported that only baseline ESR predicted LDN response and not duration of illness at baseline or severity of fibromyalgia [17,23]. Another limitation is both studies conducted in the US were restricted to women [17,18], and the study conducted in Egypt did not report gender [19].

The measurement of pain remains challenging in both clinical practice and research. Not only is pain a very personal experience, but for individuals with fibromyalgia concurrent symptoms including fatigue, poor sleep and cognitive dysfunction can be equally debilitating [26]. Many different scoring systems are currently in use including average pain daily score (ADPS), weekly average worst daily pain score (W-DPS), Brief Pain Inventory and Fibromyalgia Impact Questionnaire (FIQ) [27]. However, to date there is no consensus on patient reported outcome measures (PROMs) used in fibromyalgia research [28]. This makes comparison between different therapeutic agents challenging [[29], [30], [31]]. Several organisations have offered suggestions for a core set of PROMs that may facilitate comparison across clinical trials. These include Outcome Measures in Rheumatology Clinical Trials (OMERACT) [32,33] and Initiative on Methods, Measurement, and Pain Assessment in Clinical Trials (IMMPACT) [34]. It is important that future clinical trials among individuals with fibromyalgia use standardised outcomes to facilitate comparisons between different therapeutic strategies.

In conclusion, it is our view that larger clinical studies with appropriate statistical power and standardised outcome measures, that include both men and women from diverse ethnic groups are needed to explore the efficacy of LDN among individuals with fibromyalgia. Genome-wide association studies (GWAS) that explore the associations between SNPs and fibromyalgia phenotypes may open up new areas for research. Clinical studies that correlate biomarkers, peripheral inflammatory markers and central glial activity with the lived experiences of men and women with fibromyalgia may further elucidate the pathophysiology of this condition. Together such studies may lead to the development of more effective treatment and management strategies for those living with the chronic pain and disability associated with fibromyalgia.

## Data availability statement

Data will be made available on request.

## Declaration of interest's statement

The authors declare no competing interests.

## Funding statement

This research was supported by the National Institute for Health Research (NIHR) Applied Research Collaboration Kent, Surrey and Sussex. The views expressed are those of the author(s) and not necessarily those of the NHS, the NIHR or the Department of Health and Social Care.

## Additional information

Supplementary content related to this article has been published online at https://doi.org/10.1016/j.heliyon.2023.e15638.

## Author contributor statement

All authors made substantive contributions to the concept, design, analysis and interpretation of the data. Sarah Partridge and Lisa Quadt ran the searches and extracted the data. All authors contributed to the intellectual content of the manuscript and approved the final version for submission and are accountable for the integrity of the work.

## Role of the funding source

This research was supported by the National Institute for Health Research (NIHR) Applied Research Collaboration Kent, Surrey and Sussex. The views expressed are those of the author(s) and not necessarily those of the NHS, the NIHR or the Department of Health and Social Care
