# Supplementary material for: A systematic literature review on the clinical efficacy of low dose naltrexone and its effect on putative pathophysiological mechanisms among patients diagnosed with fibromyalgia
Source: Heliyon. 2023 Apr 19;9(5):e15638. doi: 10.1016/j.heliyon.2023.e15638 (PMC10189400; doi:10.1016/j.heliyon.2023.e15638)
Supplement: Multimedia component 1 [file mmc1.pdf]

Citation

Sarah Partridge, Monica Bolton, Lisa Quadt, Harm Van Marwijk. A systematic review of the clinical efficacy of low dose naltrexone and its effect on putative pathophysiological mechanisms among patients diagnosed with Fibromyalgia. PROSPERO 2022 CRD42022312279 Available from:
https://www.crd.york.ac.uk/prospero/display_record.php?ID=CRD42022312279

Review question

[1] Do patients with Fibromyalgia treated with low dose naltrexone have greater reduction in pain scores and greater increase in quality of life indices compared to those treated with placebo in randomised controlled trials?

[2] What differences in inflammatory markers are observed among Fibromyalgia patients compared to healthy controls?
[3] What differences in brain structure and function are observed among Fibromyalgia patients compared to healthy controls?

[4] What changes in inflammatory markers and brain structure and function are observed among Fibromyalgia patients taking low dose naltrexone?

Searches

Sources to be searched: Electronic bibliographic databases: MEDLINE, EMBASE, PsycINFO, The Cochrane Library (Cochrane Database of Systematic Reviews, Cochrane Central Register of Controlled Trials (CENTRAL), Cochrane Methodology Register). UK, European and US clinical trial registries. Reference lists from the selected papers will be cross-checked with the database search results.

Search dates: Inception to the date the searches are run. The searches will be re-run before the final analyses and further studies retrieved for inclusion.

Restrictions: English language

Types of study to be included

[1] We will include randomised controlled intervention trials of human subjects with a diagnosis of Fibromyalgia who received treatment with low dose naltrexone (4-12mg per day).

[2] We will include observational studies of human subjects with a diagnosis of Fibromyalgia for the assessment of pathophysiology.

[3] Case series and case reports will be excluded due to the high potential for bias in these study designs. Animal studies will also be excluded.

Condition or domain being studied

Patients with Fibromyalgia treated with and without low dose naltrexone.

Participants/population

Human subjects of any age with a diagnosis of Fibromyalgia (any recognised diagnostic criteria).

Intervention(s), exposure(s)

Patients with Fibromyalgia treated with oral low dose naltrexone, 4-12mg per day.

Comparator(s)/control

Patients with Fibromyalgia receiving placebo. Healthy control subjects.

Context

Fibromyalgia is a diagnosis of exclusion, when no other explanation better fits the symptoms of widespread pain, fatigue and poor sleep that patients experience. The precise aetiology of Fibromyalgia remains unknown, although interactions between genes, biology and psychosocial variables have been proposed (Üceyler 2017). Research to elucidate the pathophysiology of Fibromyalgia has led to various purported mechanisms that include inflammatory processes (Üceyler 2011) and changes in the functional connectivity between regions of the brain (Cagnie 2017). Current recommended treatments for Fibromyalgia focus on exercise, psychological therapies and antidepressants (NICE guideline NG193). Patients report these are only partially helpful (Bolton 2019). Naltrexone 50 mg once daily is currently only licensed for the treatment of drug and alcohol addiction. However, many patients with Chronic Pain Syndromes access low dose naltrexone (4 -12 mg) privately and report improvement in symptoms and quality of life (Bolton 2019). There is a need for a systematic literature review of the clinical efficacy of low dose naltrexone and its effect on putative pathophysiological mechanisms among patients diagnosed with Fibromyalgia.

REFERENCES

Üceyler et al. Schmerz 2017;31(3):239-45.

Cagnie et al. Seminars in Arthritis and Rheumatism 2017;44:68-75.

Üceyler et al. BMC Musculoskeletal Disorders 2011;12:245.

NICE guideline NG193. www.nice.org.uk/guidance/ng193.

Bolton et al. BMJ Case Rep 2020;13:e232502.doi:10.1136/bcr-2019-232502.

Main outcome(s)

Change in pain score and quality of life indices from randomisation to last available follow-up visit among patients with Fibromyalgia treated with low dose naltrexone.

Measures of effect

Average change in pain score and quality of life indices from randomisation to last available follow-up visit among patients with Fibromyalgia treated with low dose naltrexone.

Additional outcome(s)

[1] Physiological differences observed in patients with Fibromyalgia compared to healthy controls including, but not limited to, inflammatory markers and brain structure and function.

[2] Physiological changes among patients with Fibromyalgia treated with low dose naltrexone compared to placebo including, but not limited to, inflammatory markers and brain structure and function.

Measures of effect

We will summarise and compare the observed differences between patients with Fibromyalgia and healthy controls; and among patients with Fibromyalgia treated with an without low dose naltrexone

Data extraction (selection and coding)

We will follow the PRISMA guidelines. In summary we will retrieve the titles and/or abstracts of studies using our pre-defined search strategy. These will be independently reviewed by two review authors to identify studies that potentially meet the inclusion criteria outlined above. The full text of these potentially eligible studies will be retrieved and independently assessed for eligibility by two review team members. Any disagreement between them over the eligibility of particular studies will be resolved through discussion with a third reviewer. The results of the search and selection process will be documented using the PRISMA flow chart. A standardised, pre-piloted form will be used to extract data from the included studies. Two review authors will extract the data independently, discrepancies will be identified and resolved through discussion with a third author where necessary. The extracted data will be used to assess study quality and for evidence synthesis and will include: study methodology; number of patients with Fibromyalgia; participant demographics, baseline characteristics; details of the intervention and control conditions; recruitment and study completion rates; measurements and outcomes; indicators of acceptability to users; sources of

funding. We will consider the quality of evidence using the GRADE method (Grading of Recommendations Assessment, Development and Evaluation).

Risk of bias (quality) assessment

Two review authors will independently assess the risk of bias in the included studies. For the randomized controlled trials we will use the Cochrane risk of bias tool – including the additional criteria of number of trial participants (trial size). For the mechanistic studies we will consider how patients and control subjects were recruited to the study, the inclusion and exclusion criteria, the completeness of the reported outcome data, how missing/incomplete data was dealt with in the analyses, what sensitivity analyses were performed. We will consider if participant exclusions, attrition and incomplete outcome data were adequately addressed in the published report. We will consider how selective outcome reporting may have affected the study result. Disagreements between the review authors over the risk of bias in particular studies will be resolved by discussion with involvement of a third review author where necessary. We will analyse recommendations offered for mechanistic pathways in the papers we review.

Strategy for data synthesis

We will provide a narrative (descriptive) synthesis of the findings from the included studies, structured around the inclusion criteria, investigations performed and outcome measures reported. We anticipate that there will be limited scope for meta-analysis because of the expected small number of existing trials in patients with Fibromyalgia. We will consider publication bias by reporting the number of trials that report no observed differences between treatment and control groups.

Analysis of subgroups or subsets

This is a qualitative synthesis and while subgroup analyses may be undertaken it is not possible to specify the groups in advance.

Contact details for further information

Dr Sarah Partridge
s.partridge@bsms.ac.uk

Organisational affiliation of the review

Brighton and Sussex Medical School
www.bsms.ac.uk

Review team members and their organisational affiliations

Dr Sarah Partridge. Brighton and Sussex Medical School
Dr Monica Bolton. Brighton and Sussex Medical School
Dr Lisa Quadt. Brighton and Sussex Medical School
Professor Harm Van Marwijk. Brighton and Sussex Medical School

Collaborators

Dr Charlie Thompson. Brighton and Sussex Medical School
Dr Jessica Eccles. Brighton and Sussex Medical School
Dr Alessandro Colasanti. Brighton and Sussex Medical School
Professor Stephen Bremner. Brighton and Sussex Medical School
Dr Chris Jones. Brighton and Susssex Medical School
Dr Karin Due Bruun. University of Southern Denmark

Type and method of review

Intervention, Narrative synthesis, Systematic review

Anticipated or actual start date

01 March 2022

Anticipated completion date [1 change]

31 August 2022

Funding sources/sponsors

Brighton and Sussex Medical School

Conflicts of interest

Language
English

Country
England

Stage of review [1 change]

Review Completed not published

Subject index terms status
Subject indexing assigned by CRD

Subject index terms
Fibromyalgia; Humans; Naltrexone; Narcotic Antagonists; Treatment Outcome

Date of registration in PROSPERO
24 February 2022

Date of first submission
22 February 2022

Stage of review at time of this submission [1 change]

**Stage**	**Started**	**Completed**
Preliminary searches	Yes	Yes
Piloting of the study selection process	Yes	Yes
Formal screening of search results against eligibility criteria	Yes	Yes
Data extraction	Yes	Yes
Risk of bias (quality) assessment	Yes	Yes
Data analysis	Yes	Yes

Revision note
17/08/2022 update to record to report the review has been completed and the manuscript is being prepared for journal submission

The record owner confirms that the information they have supplied for this submission is accurate and complete and they understand that deliberate provision of inaccurate information or omission of data may be construed as scientific misconduct.
The record owner confirms that they will update the status of the review when it is completed and will add publication details in due course.

Versions
24 February 2022
